# Machine learning prediction of multiple anthelmintic resistance and gastrointestinal nematode control in sheep flocks

**DOI:** 10.1590/S1984-29612024014

**Published:** 2024-03-18

**Authors:** Simone Cristina Méo Niciura, Guilherme Martineli Sanches

**Affiliations:** 1 Embrapa Pecuária Sudeste, São Carlos, SP, Brasil; 2 Departamento de Ciência do Solo, Escola Superior de Agricultura “Luiz de Queiroz” - ESALQ, Universidade de São Paulo - USP, Piracicaba, SP, Brasil

**Keywords:** CARTs, machine learning, multidrug resistance, gastrointestinal nematodes, random forest, CARTs, aprendizado de máquina, resistência múltipla, nematoides gastrintestinais, random forest

## Abstract

The high prevalence of *Haemonchus contortus* and its anthelmintic resistance have affected sheep production worldwide. Machine learning approaches are able to investigate the complex relationships among the factors involved in resistance. Classification trees were built to predict multidrug resistance from 36 management practices in 27 sheep flocks. Resistance to five anthelmintics was assessed using a fecal egg count reduction test (FECRT), and 20 flocks with FECRT < 80% for four or five anthelmintics were considered resistant. The data were randomly split into training (75%) and test (25%) sets, resampled 1,000 times, and the classification trees were generated for the training data. Of the 1,000 trees, 24 (2.4%) showed 100% accuracy, sensitivity, and specificity in predicting a flock as resistant or susceptible for the test data. Forage species was a split common to all 24 trees, and the most frequent trees (12/24) were split by forage species, grazing pasture area, and fecal examination. The farming system, Suffolk sheep breed, and anthelmintic choice criteria were practices highlighted in the other trees. These management practices can be used to predict the anthelmintic resistance status and guide measures for gastrointestinal nematode control in sheep flocks.

## Resumo

A criação de ovinos é afetada pela alta prevalência de *Haemonchus contortus* e pela resistência anti-helmíntica, cujas complexas inter-relações podem ser elucidadas por técnicas de aprendizado de máquina. Árvores de classificação identificaram a importância de práticas de manejo em 27 rebanhos ovinos, para a predição da resistência anti-helmíntica múltipla. A resistência a cinco anti-helmínticos foi determinada pelo teste de redução de contagem de ovos nas fezes (TRCOF), e as 20 propriedades com eficácia inferior a 80% no TRCOF para quatro ou cinco anti-helmínticos foram consideradas resistentes. Os dados foram aleatoriamente separados em 1.000 amostragens de treinamento (75%) e teste (25%), e as árvores de classificação foram geradas para os dados de treinamento. Dessas, 24 árvores (2,4%) apresentaram acurácia, sensibilidade e especificidade de 100% para a predição do estado de resistência nos dados de teste. O tipo de forragem foi detectado em todas as árvores, enquanto a área de pastagem e a realização de exame de fezes foram observadas nas árvores mais frequentes (12/24). O sistema de produção, a raça ovina Suffolk e o critério de escolha do anti-helmíntico geraram quebras nas demais árvores. Essas práticas de manejo podem predizer o estado de resistência anti-helmíntica em rebanhos e orientar medidas, visando ao controle da verminose em ovinos.

## Introduction

The economic importance of sheep in the supply of animal-origin proteins mainly relies on the use of small areas unsuitable for cattle production and agriculture (Sargison, 2012). According to recent estimates, Brazilian sheep flocks, composed of 20.5 million heads (IBGE, 2022), cannot meet the national consumer demand, which depends on lamb importation (FAO, 2022). Therefore, it is necessary to support, develop, and invest in sheep production systems in Brazil to increase productivity and profitability.

In tropical countries, the main challenges in sheep farming are adverse climatic conditions, competition for resources, including water and food, and the high prevalence of gastrointestinal nematodes (McManus et al., 2011). Among them, *Haemonchus contortus* is the most prevalent and pathogenic parasite in sheep (Waller, 2004), leading to losses estimated at US$ 107.5 million per year (Chagas et al., 2022). These losses are secondary to reduced growth and weight gain, inferior meat and wool quality, high costs of therapeutics, and animal death, especially in the case of resistance to anthelmintics used for nematode control (Costa et al., 2007; Miller et al., 2012).

Despite of the large number of commercial products, only four classes of broad-spectrum anthelmintics, i.e., benzimidazoles, imidazothiazoles, salicylanilides, and macrocyclic lactones, are available for treating sheep in Brazil (Chagas et al., 2013). Free access, ease of use, and lack of technical guidance have led to massive and incorrect use of anthelmintics, reducing their efficacy and promoting multidrug resistance (Doyle & Cotton, 2019). Because anthelmintic resistance leads to treatment failure, it can be detected *in vivo* through a fecal egg count reduction test (FECRT) after anthelmintic treatment of sheep hosts (Coles, 2005).

The establishment of resistance is affected by management practices used in flocks (Barger, 1997). Thus, resistance can be delayed by reducing pasture contamination and sheep infection not only through the use of anthelmintics but also by controlling environmental conditions (Bath, 2011; Leathwick, 2012; McBean et al., 2016; McFarland et al., 2022).

Similarly to other health traits, resistance data are complex, unbalanced, and contain missing values, resulting in a nonlinear complex interrelationship between the response and explanatory variables (Speybroeck, 2012). Thus, statistical and machine learning tools are suitable for detecting the factors that lead to anthelmintic resistance. Among them, classification and regression trees (CARTs) are non-parametric techniques that explore the interactions among variables, remove irrelevant covariates, produce visual results that are easy to interpret, and can predict the response to new observations (Speybroeck, 2012; Izbicki & Santos, 2020). A classification tree is built with recursive partitioning of the explanatory variables (resulting in nodes), minimizing the errors (the lower the error, the larger the branches and the importance of the variable to predict the outcome), and explaining, as much as possible, the categorical response variable in the leaves (Speybroeck, 2012; Izbicki & Santos, 2020). The random forest approach aggregates a collection of random decision trees, aiming to optimize a predictor and explore all possible tree predictors simultaneously, usually resulting in better performance (Genuer & Poggi, 2019).

Recently, machine-learning techniques have been used in veterinary parasitology. They were used to predict the resistance of sheep against gastrointestinal nematodes (Freitas et al., 2023b), to classify Famacha score anemia based on automatic analysis of ocular conjunctiva images (Freitas et al., 2023a), to perform fecal Strongyle egg counts from video footage (Bucki et al., 2023), to detect environmental risk factors affecting haemonchosis incidence based on egg per gram counts (Suresh et al., 2022), and as a potential approach for anthelmintic drug discovery (reviewed by Zamanian & Chan, 2021). To the best of our knowledge, this is the first study to use machine learning to predict anthelmintic resistance in flocks based on management practices.

In a previous study using the same data (Niciura et al., 2012), risk factors in management practices were identified using the frequency of genetic resistance to benzimidazoles in *H. contortus* as the response variable. However, as most flocks were resistant to other anthelmintic classes besides benzimidazoles, using FECRT data as the response variable and applying machine learning approaches have the potential to identify the factors involved and their interrelationships with the establishment of multiple anthelmintic resistance.

Thus, knowledge of parasite epidemiology, resistance status, and management practices in flocks can be used to develop new strategies for parasite control. In this study, classification trees were built based on 36 management practices and FECRT efficacy data for five anthelmintics (albendazole, closantel, ivermectin, levamisole, and moxidectin) in 27 sheep flocks in São Paulo State, Brazil. The objective of this study was to generate classification trees and identify relevant management practices that lead to resistance to multiple anthelmintics.

## Material and Methods

A survey was conducted among 34 sheep farmers in São Paulo State, Brazil, from 2008 to 2010 to obtain information on 36 management practices related to infrastructure, health, and feed management (Veríssimo et al., 2012). In addition, an FECRT for a control group and five anthelmintics (albendazole, closantel, ivermectin, levamisole, and moxidectin) assessed the reduction in fecal egg counts (FEC) (Ueno & Gonçalves, 1998) 10-14 days after treatment, obtaining anthelmintic efficacy in 27 flocks (Veríssimo et al., 2012). The following formula was used for calculation of anthelmintic efficacy in FECRT:


$$\%Efficacy_{AH}=\;\frac{meanFEC_{Control}+meanFEC_{AH}}{meanFEC_{Control}}\;x\;100$$ (1)

where Efficacy_AH_ is the efficacy of each anthelmintic; meanFEC_Control_ is the mean fecal egg count for the non-treated control group 10-14 days after FECRT; and meanFEC_AH_ is the mean fecal egg count for each anthelmintic-treated group 10-14 days after FECRT. An anthelmintic efficacy lower than 80% in FECRT indicate high resistance (Voigt et al., 2022).

In total, 1,000 classification trees were built from the 27 sheep flock dataset to predict the development of *in vivo* multidrug resistance (as the categorical response variable) based on management practices (explanatory variables) used in each flock. Considering the quartile distribution of anthelmintics with high resistance, flocks were classified as resistant (1) when presenting 4 and 5 anthelmintics with FECRT < 80% (20 flocks), and as susceptible (0) when presenting 0 to 3 anthelmintics with FECRT < 80% (7 flocks).

All quantitative (total area, grazing pasture area, time in farming, number of dams, number of sires, and number of heads) and categorical (wetland, flooring, main income source, secondary exploration, grazing of cattle, farm accounting, technical assistance, frequent animal incorporation, quarantine, Dorper, Ile de France, Santa Inês, Texel and other breeds, crossbred, region of animal origin, forage species, rotational grazing, farming system, shared grazing, anthelmintic choice, anthelmintic rotation, combination of drugs, deworming schedule, dose-and-move practice, estimation of weight, FEC examination, Famacha, FECRT, and resistance status) variables used in the analysis and their descriptive statistics in the 27 flocks are presented in Tables 1 and 2.

**Table 1:** Descriptive statistics for quantitative variables of management practices and anthelmintic resistance in 27 sheep flocks from São Paulo State, Brazil.

**Variable**	**Min**	**1^st^ Quart**	**Median**	**Mean**	**3^rd^ Quart**	**Max**	**NA**
Total area (ha)	7.5	33.0	100.0	273.4	217.0	2000.0	2
Grazing pasture area (ha)	2.0	9.5	27.2	49.0	63.2	194.0	3
Time in farming (years)	2.0	4.3	5.0	8.6	8.0	51.0	1
Number of dams	50.0	133.2	241.0	397.8	450.0	1500.0	3
Number of sires	1.0	4.0	12.5	46.1	22.5	773.0	3
Number of heads	95.0	183.0	316.0	594.1	742.0	2028.0	2
Anthelmintics with FECRT < 80%	1.0	3.5	4.0	3.9	5.0	5.0	0

FECRT = fecal egg count reduction test; Quart = quartile; Min = minimum; Max = maximum; NA = missing values.

**Table 2:** Descriptive statistics for categorical variables of management practices and anthelmintic resistance in 27 sheep flocks from São Paulo State, Brazil.

**Variable**	**Category 1**	**Category 2**	**Category 3**	**Category 4**	**Category 5**	**Category 6**	**Category 7**
Wetlands	17 (no)	10 (yes)					
Flooring	24 (concrete)	2 (slat)	1 (NA)				
Main income source	19 (no)	8 (yes)					
Secondary exploration	16 (no)	9 (reproduction)	1 (wool)	1 (milk)			
Grazing of cattle	17 (yes)	10 (no)					
Farm accounting	16 (yes)	11 (no)					
Technical assistance	13 (frequent)	6 (sporadic)	8 (no)				
Frequent animal incorporation	21 (no)	6 (yes)					
Quarantine	21 (yes)	5 (no)	1 (NA)				
Dorper breed	19 (no)	7 (yes)	1 (NA)				
Ile de France breed	21 (no)	6 (yes)					
Santa Inês breed	22 (yes)	5 (no)					
Suffolk breed	17 (no)	10 (yes)					
Texel breed	20 (no)	7 (yes)					
Other breeds	22 (no)	5 (yes)					
Crossbred	14 (yes)	12 (no)	1 (NA)				
Region of animal origin	13 (SE)	6 (NE)	4 (SE + NE)	1 (SE + CW)	1 (SE + S)	1 (NE + S)	1 (NA)
Forage species	7 (*Brachiaria*)	6 (*Cynodon*)	5 (*Panicum*)	3 (*Brachiaria* + *Panicum*)	3 (*Brachiaria* + *Panicum* + *Cynodon*)	3 (*Brachiaria* + *Panicum* + *Cynodon* + Pulse)	2 (other)
Rotational grazing	22 (yes)	5 (no)					
Farming system	22 (semi-intensive)	3 (intensive)	2 (extensive)				
Shared grazing	14 (no)	7 (cattle)	5 (horses + cattle)	1 (horses)			
Anthelmintic choice	15 (technician)	4 (seller)	3 (efficacy)	2 (other)	1 (technician + efficacy)	1 (experience)	1 (NA)
Anthelmintic rotation	11 (based on efficacy)	8 (after each treatment)	5 (based on FECRT)	3 (NA)			
Combination of drugs	17 (no)	10 (yes)					
Deworming schedule	8 (target + strategic)	7 (target)	6 (fixed)	3 (fixed + target + strategic)	1 (strategic)	1 (fixed + strategic)	1 (fixed + target)
Dose-and-move practice	16 (no)	11 (yes)					
Estimation of weight	16 (visual)	8 (scale)	3 (scale + visual)				
FEC examination	11 (sporadic)	11 (no)	3 (monthly)	1 (annual)	1 (quarterly)		
Famacha	18 (yes)	9 (no)					
FECRT	22 (no)	5 (yes)					
Resistance status	20 (resistant)	7 (susceptible)					

FEC = fecal egg counts; FECRT = fecal egg count reduction test; NA = missing information; SE = Southeast; NE = Northeast; CW = Center-West; S = South.

The data were split into 1,000 random training (75%) and test (25%) sets, and trees were built using raw data (Steinberg, 2009) with the recursive partitioning (*rpart*) package (Therneau & Atkinson, 2022) in R version 4.2.1 (R Core Team, 2022). The parameters included method = “class”, split = “gini”, cp = 0, minsplit = 1, maxdepth = 30, and xval = 10, and the trees were plot with treemisc (Greenwell, 2022). The accuracy of prediction was calculated from the confusion matrix, and the package *caret* (Kuhn, 2022) was used to calculate the area under the receiver operating characteristic (ROC) curve (AUC), sensitivity, and specificity.

In addition, a random forest approach was used to confirm the results obtained from the classification trees. To this aim, missing data were replaced with the mean (for quantitative variables) or mode (for categorical variables). Then, a random forest (Liaw & Wiener, 2002) model, including the parameters importance = T, proximity = T, ntrees = 1000, and sample size was fitted to the training data (75%) and validated using the test data (25%).

## Results

**Figure 1:**
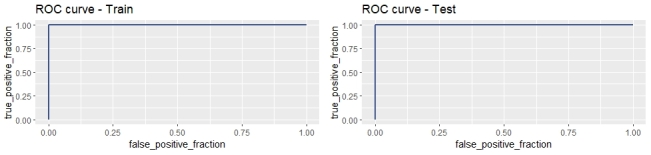
Area under the receiver operating characteristic (ROC) curve for prediction of multiple anthelmintic resistance in 27 sheep flocks with classification trees using training (75%) and test (25%) data.

**Figure 2:**
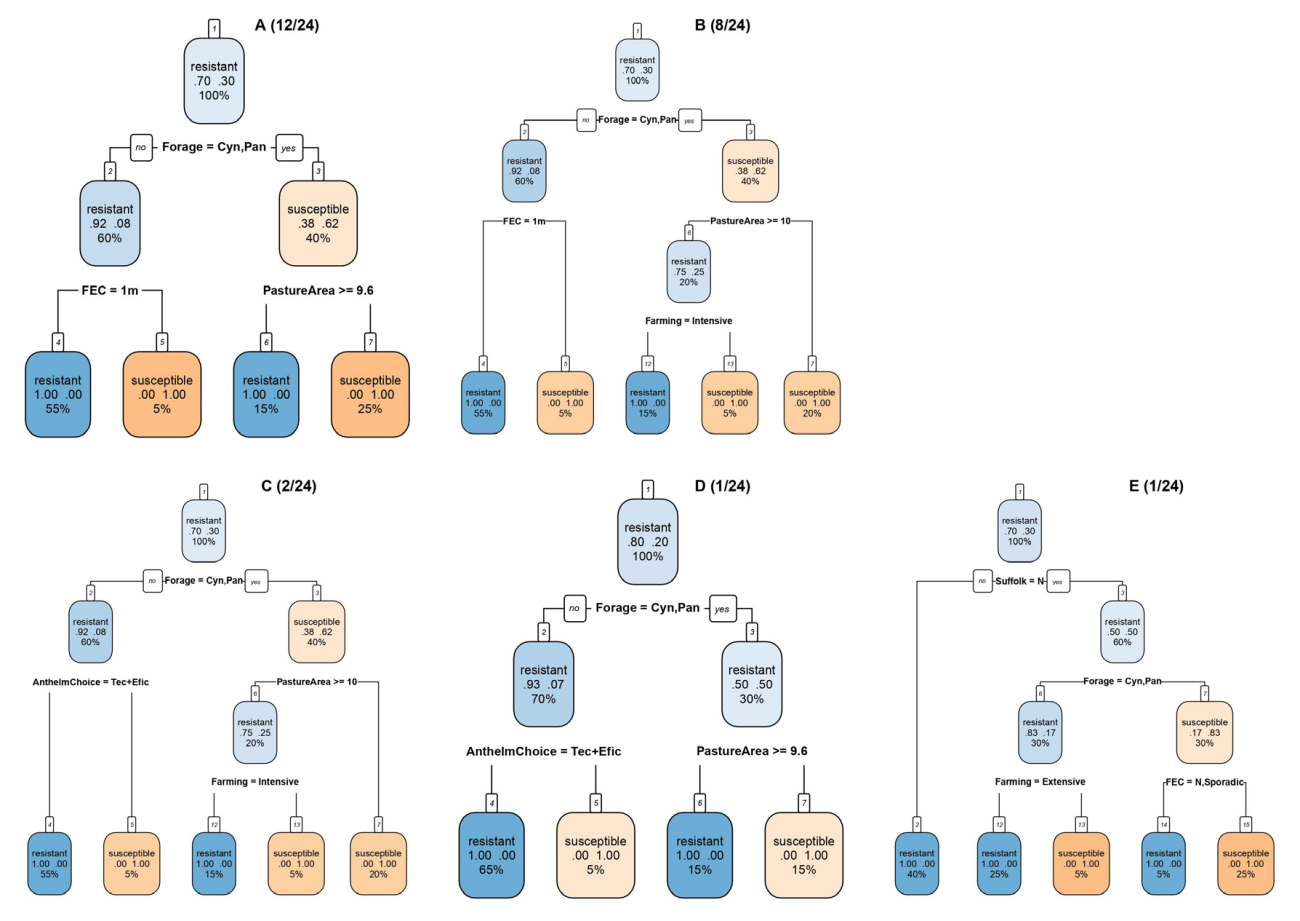
Classification trees of multiple anthelmintic resistance based on forage species, fecal egg count exam (FEC), grazing pasture area in ha, anthelmintic choice criteria, and Suffolk sheep breed in flocks of São Paulo State, Brazil. Cyn: *Cynodon*, Pan: *Panicum*, 1m = monthly, Tec+Effic = decision for anthelmintic treatment based on technical recommendation and efficacy.

From the 1,000 classification trees fitted to the training data, 24 trees predicted the anthelmintic resistance status in the test group with 100% accuracy, sensitivity, and specificity and an AUC of 1, and the ROC curves for training and test data are shown in Figure 1. Forage species, grazing pasture area, and FEC were the explanatory variables predicting multidrug resistance in the most frequent (12/24) trees (Figure 2A). According to these trees, farms that have an exclusive pasture of *Cynodon* or *Panicum* forage species (right branch) and a grazing pasture area ≥ 9.6 ha are susceptible, whereas, when other forage species (left branch) are present, they are susceptible only if they perform FEC examination every month (Figure 2A). In the second most frequent trees (8/12) (Figure 2B), the farming system was included in addition to forage species, FEC exams, and pasture grazing areas. Farms with pasture areas < 10 ha are susceptible only if they adopt intensive farming systems. Other management practices were detected in the less frequent trees, such as anthelmintic choice criteria based on both technical recommendations and efficacy, resulting in susceptibility (Figures 2C and 2D), and raising the Suffolk sheep breed, resulting in anthelmintic resistance (Figure 2E).

Among the 24 classification trees, 24 out of the 36 investigated management practices were identified as important for the prediction of anthelmintic resistance. The importance scores were then retrieved, and the variables were ordered based on the sum of their scores (Table 3), revealing the grazing pasture area, forage species, number of heads, number of dams, and FEC examination as the top five variables for the prediction of multidrug resistance.

**Table 3:** Sum of scores for important management practices for classification trees built to predict anthelmintic resistance in sheep flocks.

**Variable**	**Importance score sum**
Pasture area	80.56
Forage species	69.23
Number of heads	50.66
Number of dams	40.45
FEC exam	39.09
Total area	35.93
Region of animal origin	29.08
Deworming schedule	28.75
Time in farming	21.00
Farming system	16.87
Combination of anthelmintics	12.83
Shared grazing with other species	12.69
Famacha exam	10.51
Dose-and-move practice	9.82
Grazing of cattle	8.86
Anthelmintic choice	8.61
Number of sires	8.19
Santa Ines breed	6.57
Texel breed	5.26
Frequent animal incorporation	3.45
Main income source	3.00
Suffolk breed	2.40
FECRT	1.06
Technical assistance	0.60

**Figure 3:**
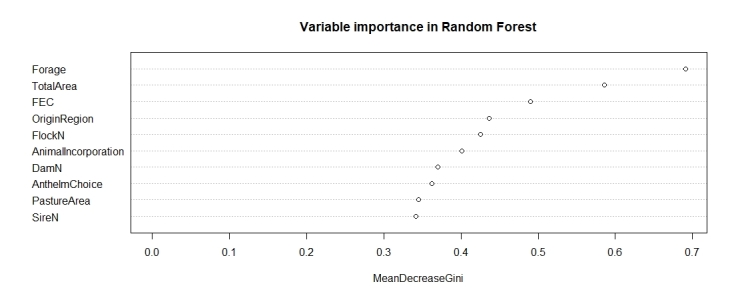
Importance of management practice variables based on the random forest model to predict anthelmintic resistance in sheep flocks.

The random forest model resulted in a prediction with 86% accuracy, 100% sensitivity, and 50% specificity. Despite the inferior results compared to the classification trees, the ten most important variables (Figure 3), based on node impurities, are among those highlighted in the trees.

## Discussion

In well-managed flocks, fewer unnecessary treatments are applied to animals; consequently, selective pressure on the development of anthelmintic resistance is reduced. In this study, several management practices applied to sheep flocks were able to predict anthelmintic resistance status. The most important practices highlighted in the classification trees were forage species, grazing pasture area, FEC examination, farming system, criteria for anthelmintic choice, and Suffolk sheep breed.

Higher anthelmintic susceptibility was detected in farms with exclusive pastures of *Cynodon* and *Panicium*, whereas the combination of these species with other forage and pulse species resulted in resistance. Thus, handling only one type of forage may be easier than handling multiple different exigencies. Additionally, the forage itself may influence anthelmintic resistance in several ways. First, short-cropped grass accumulates more concentrated larvae than taller grass (Bath, 2011). Second, forage nutritional quality influences animal nutrition and physical condition, which, in turn, affects the effectiveness of the immune response and mechanisms to support the parasite load (reviewed by Walkden-Brown & Kahn, 2002; reviewed by McRae et al., 2015). *Brachiaria humidicola* and *Panicum maximum* cv. Massai had low alimentary values, whereas *B. brizantha* cv. Marandu had a high forage quality and *Cynodon* cv. Coastcross and *P. maximum* cv. Colonião, Tanzânia, and Tobiatã presented high nutritional and alimentary values (Corrêa & Santos, 2009). Nutritional variations among forage species may have influenced the response of sheep to nematodes and infection levels. Additionally, the morphology and structure of plants interfere with nematode larval migration from the feces in the soil to the forage tip ingested by animals and larval survival as a consequence of solar radiation exposure and desiccation. Seasonal temperature and humidity conditions affect *H. contortus* larval migration in *B. decumbens* (Santos et al., 2012), and a large number of infective larvae were recovered from *U. humidicola* and *M. maximus* cv. Massai compared with *U. brizantha* cv. Marandu and *M. maximus* cv. Mombaça (Pires et al., 2021). However, no differences in larval migration were observed among the *Stylosanthes* spp., *B. brizantha* cv. Marandu, *B. brizantha* cv. Xaraes, and *P. maximum* cv. Tanzânia (Oliveira et al., 2009; Urzedo et al., 2022). Thus, the availability of larvae in pastures being ingested by the animals and affecting infection rates may be influenced not only by the species (which was the information retrieved in the surveys) but also by forage cultivars.

In sheep grazing pasture areas, anthelmintic susceptibility associated with larger areas can be related to the increase in food supply and consequent improved animal nutrition, dilution of nematodes in the pasture reducing sheep infection, and maintenance of refugia. Refugia is a unanimous management practice used for the control of anthelmintic resistance. It consists of retaining part of the parasite population in animals and environment not exposed to anthelmintics, assuring the stock of genes of susceptibility (Kenyon et al., 2009).

Monthly fecal examination was associated with anthelmintic susceptibility, probably because monitoring animal infection can be used as an effective decision criterion for target-selective treatment (Kenyon et al., 2009). Additionally, the criteria used for anthelmintic choice influenced resistance, with beneficial effects observed when the decision was based on technical and efficiency criteria (through FECRT) in contrast to the seller, price, or farmer’s experience.

Farming systems affected resistance, with intensive farming resulting in lower resistance in smaller pasture areas. In intensive systems, higher stock rate and animal concentration may increase contamination. However, better pastures, feeding supplementation, and confinement practices may improve the nutritional conditions of animals, leading to greater resistance to worms (Van Wyk, 1990).

Raising Suffolk sheep was a practice associated with resistance in the classification trees. There are differences in parasite resistance among sheep breeds, and Suffolk shows higher susceptibility to gastrointestinal nematodes than Santa Inês (Amarante et al., 2004). Thus, the selection of more resistant breeds or individuals within breeds is an effective strategy to reduce anthelmintic use in flocks and delay the establishment of resistance (Zvinorova et al., 2016).

Other variables were also identified as important for the prediction of anthelmintic resistance. However, because they were not highlighted in the classification tree nodes, their effects may be distinct based on their interactions with other variables. For example, sheep grazing pastures shared with other animal species have been used for gastrointestinal nematode control. Since most parasites are species-specific, shared grazing with cattle, horses, or pigs can reduce environmental contamination with nematodes (Barger, 1997; Forbes, 2021); however, as an adverse effect, co-grazing sheep with cattle may accelerate the development of anthelmintic resistance, as it may reduce the number of parasites in refugia on pasture (see Falzon et al., 2014 for a review).

Other practices were related to farm structure and technical level, including the number of dams, heads, and sires; total area; cattle grazing; and sheep production as the main income source. These practices reflect farm production levels, care given to sheep, the availability of animals for selection based on parasite susceptibility, and pasture area. Time spent in sheep farming may also have a dual effect; while farming for very short periods could be associated with the use of pastures that are less contaminated with larvae, farming for longer periods may result in learning gains to control the parasites.

Other important practices were associated with anthelmintic treatment protocols. Target-selective and strategic treatments are usually preferred over fixed deworming schedules, but refugia, parasitic load, and animal physical condition should be considered when choosing a deworming protocol (Bath, 2011). Anthelmintic combinations promote nematode control in the presence of single or multiple resistance and slow the development of resistance under certain prerequisites. However, resistance to all drugs used in combination can occur if refugia are insufficient or if resistance is high (reviewed by Bartram et al., 2012). Famacha examinations detect anemia, identify animals for target-selective treatment, and reduce anthelmintic usage, but only when *H. contortus*, which is hematophagous, is the most prevalent species in the flock (Bath, 2011). Dose-and-move, also known as drench-and-shift, involves moving animals after treatment to a new clean pasture and is considered a high-risk practice for anthelmintic resistance, as it reduces refugia due to contamination of the new pasture only with resistant parasites (Waghorn et al., 2009).

Replacement of the parasite population can occur through the import of animals from other farms; however, the consequences depend on the resistance status of these parasites. Thus, frequent animal incorporation and the region of animal origin can increase anthelmintic resistance when imported helminths are resistant to anthelmintics (Bath, 2011), but the opposite may occur if susceptible worms are imported.

Interestingly, trees provided management alternatives; if a farmer could not modify one practice, it was possible to implement a different strategy to reduce anthelmintic resistance. For example, if changing the forage species of the pasture is impossible, monitoring the fecal egg counts of animals every month can reduce anthelmintic resistance, and the use of intensive farming can compensate the reduced availability of pasture areas in farms. However, as intensification increases not only stocking rate, but also parasitism levels (Waller, 2006), strategies for environmental decontamination and reduction of larvae in pasture, such as using integrated crop-livestock systems (Almeida et al., 2018) or biological control with nematophagous fungi (reviewed by Waller, 2006), may increment the integrated control of gastrointestinal nematodes in sheep flocks.

Classification trees provide a visual interpretation of data and require less computational memory, but are more prone to overfitting and instability, and small perturbations in the dataset can lead to large variations in predictions (Fratello & Tagliaferri, 2019). Due to this disadvantage, a random forest model was also evaluated. Random forest approaches, despite requiring higher computational memory and missing the easy interpretation of the trees, aggregate independent classifiers (or independent trees) and usually result in better performance (Fratello & Tagliaferri, 2019). In the present study, despite selecting almost the same variables of importance, better prediction results were obtained with the classification trees than with the random forest model. Considering the small sample size (27 observations) and unbalanced data (20 resistant and 7 susceptible flocks), building 1,000 classification trees with cross-validation after altering the training set (resampling) resulted in trees with higher prediction accuracy compared to the random forest results, which was based on the aggregation of 1,000 independent trees, but only after one subset of the database.

## Conclusions

Machine learning tools are suitable for the fast, easy, noninvasive, and less costly identification and classification of response patterns and their complex relationship to determinant factors. The development of classification trees to predict anthelmintic resistance in sheep flocks based on management practices could provide alternatives for controlling the global multidrug resistance problem. Practices such as the exclusive use of forage species in pastures, monthly FEC examination of animals, intensive farming systems, pasture area available for grazing, and the use of parasite-resistant sheep breeds can be implemented to delay the establishment of anthelmintic resistance in flocks. Furthermore, prioritizing management changes based on variable importance is a smart decision made by farmers and technicians to promote sheep health and nematode control, reduce the use of anthelmintics, and manage the establishment of anthelmintic resistance.
